# Retrospective Study on Acute Kidney Injury among Cholera Patients in an Outbreak in Whitefield, Bengaluru

**DOI:** 10.1155/2021/6682838

**Published:** 2021-06-04

**Authors:** Girish P Vakrani, Tanuja Nambakam

**Affiliations:** ^1^Department of Nephrology, Vydehi Institute of Medical Sciences and Research Centre, #82, EPIP Area, Whitefield, Bengaluru 560066, Karnataka, India; ^2^Department of General Medicine, Vydehi Institute of Medical Sciences and Research Centre, #82, EPIP Area, Whitefield, Bengaluru 560066, Karnataka, India

## Abstract

**Introduction:**

Cholera is gastroenteritis caused by *Vibrio cholerae*. It presents with vomiting, severe secretory diarrhoea, and dehydration. It can cause severe complications with severe electrolyte imbalances and oligoanuric acute kidney injury due to acute tubular necrosis secondary to dehydration or infection itself. However, cholera presenting with significant proteinuria and acute kidney injury has not been reported. Hence, this study was conducted. *Aims and Objectives.* This aim of this study was to assess clinical features, treatment, and prognosis of AKI in cholera patients; to correlate proteinuria with AKI in cholera patients; and to compare cholera patients with normal kidney function and those with AKI. *Material and Methods*. It was a retrospective observational study involving patients with cholera. Information regarding cholera patients with acute kidney injury, proteinuria, and prognosis were collected.

**Results:**

Most of the patients had significant vomiting, moderate-to-severe diarrhoea, dehydration, and hypovolaemic shock. Cholera caused severe complications such as severe electrolyte imbalances including hyponatraemia and hypokalaemia, acute kidney injury, and proteinuria secondary to dehydration or infection. A surprising finding noted was the lack of significant association between the onset of acute kidney injury and usual risk factors such as hypovolaemic shock and dehydration. It was found that proteinuria had influenced the onset of acute kidney injury, but it did not influence recovery. As there was complete recovery in kidney function, none of the cases required kidney biopsy. There was no mortality noted.

**Conclusions:**

This study points towards the rare occurrence of proteinuria and acute kidney injury in *Vibrio cholerae* infection with spontaneous remission of kidney disease with appropriate therapy.

## 1. Introduction

Cholera is gastroenteritis caused by *Vibrio cholerae*. It presents with vomiting, severe secretory diarrhoea, and dehydration. It presents as watery, rice-water stools with a fishy odour. It can cause severe complications with severe electrolyte imbalances such as hyponatraemia, hypernatraemia, and hypokalaemia and oligoanuric acute kidney injury (AKI) due to acute tubular necrosis (ATN). Renal complications such as AKI, metabolic acidosis, ATN, and acute tubulointerstitial nephritis (ATIN) secondary to dehydration or infection itself have been reported [1]. However, cholera presenting with significant proteinuria and acute kidney injury has not been reported. Even there is a paucity of data on cholera presenting with AKI. Hence, this study was conducted.

## 2. Aims and Objectives

1. To assess clinical features, treatment, and prognosis of AKI in cholera patients
2. To correlate proteinuria (urine protein-creatinine ratio) with AKI (serum creatinine) in cholera patients
3. To compare cholera patients with normal kidney function and those with AKI

### 2.1. Description

This retrospective observational study was conducted to look into factors determining the onset of AKI in cholera and its prognosis.

## 3. Material and Methods

### 3.1. Study Design

It is a retrospective observational study.

### 3.2. Study Subjects

#### 3.2.1. Inclusion Criteria

This study included patients diagnosed with cholera with stool hanging drop preparation in Vydehi Hospital.

#### 3.2.2. Exclusion Criteria

Patients with previously established kidney failure were excluded from the study.

### 3.3. Study Setting and Study Population

#### 3.3.1. Study Area

This study was conducted using medical records of cholera-diagnosed patients at Vydehi Institute of Medical Sciences and Research Centre, which is a tertiary care hospital in Bangalore, during outbreak occurred during March–April 2020 after taking ethical clearance.

### 3.4. Data Collection

Data were collected regarding the patient's demography, clinical features, laboratory parameters, and treatment details. Information pertaining to the demographics of the patients such as age and gender were collected along with the history of comorbidities such as diabetes mellitus and hypertension. Data regarding the severity of diarrhoea, urine output, vital parameters such as pulse and blood pressure, and biochemical parameters such as complete blood cell count, serum electrolytes, blood urea, serum creatinine, urine analysis, urine protein-creatinine ratio, abdominal ultrasound, stool for hanging drop preparation, and treatment details were collected.

### 3.5. Definitions/Severity of Clinical Entities

#### 3.5.1. Diarrhoea

- Mild (few diarrhoea stools/day)
- Moderate (more than few diarrhoea stools but <10 times/day)
- Severe (diarrhoea stools >10 times/day) [2]

#### 3.5.2. Dehydration

- Moderate dehydration (irritable, sunken eyes, thirsty, skin goes back slowly on skin pinch: >2/4)
- Severe dehydration (lethargic, sunken eyes, drinking poorly, skin goes back very slowly on skin pinch: >2/4) [3]

#### 3.5.3. Urine PCR

  >0.2 is abnormal
   >2 is nephrotic proteinuria [4]

#### 3.5.4. AKI Definition

- Serum creatinine increases by 3 times, or
- Urine output (oliguria: <400 ml/day or <0.3 ml/kg/hr for 24 hours) [5]

Basal serum creatinine was referred as 0.9–1.3 mg/dl (males) and 0.6–1.1 mg/dl (females). If basal values were not available, 3 times increase from the first value was taken into account.

### 3.6. Data Analysis and Statistical Analysis

The data were collected, entered, and analysed with SPSS version 19. Continuous variables were presented as mean ± standard deviation or minimum, maximum, Q1, Q2, and Q3. Categorical variables were presented as frequency and percentage. Chi-squared test or Fisher's exact test was performed to see the association between any two categorical variables.

### 3.7. Human Subjects Protection

The information of subjects was kept confidential.

### 3.8. Time Frame

Data collection was started after obtaining the ethics committee/IRB's approval.

### 3.9. Expected Results and Benefits

It was expected that good information regarding the cholera patients, the nature of their comorbidities, the basic cause of AKI, and the incidence of complications such as proteinuria, AKI, and the prognosis will be collected. It would also help obtain information on any risk factors and comorbidities influencing the outcome of cholera diarrhoea. The study will give information regarding the cholera-induced renal disease.

## 4. Results

Among total enrolled 55 cholera patients, 32years was the mean age at presentation; 40 (72.7%) were male; 51 (92.7%) had a history of food poisoning; 6 (10.9%) had a history of antacid intake; and 4 (7.3%) had diabetes mellitus. Regarding the severity of diarrhoea, 31 (56.4%) had severe diarrhoea; 21 (38.2%) had moderate diarrhoea; and 3 (5.5%) had mild diarrhoea. It is noted that 51 (92.7%) had vomiting; 31 (56.4%) had abdominal pain; 8 (14.5%) had fever; 16 (29.1%) had blood in stools; 52 (94.5%) had hypovolaemic shock; 52 (94.5%) had severe dehydration; 19 (34.5%) had microscopic haematuria; 43 (78%) had AKI; 54 (98.2%) had normal kidneys in ultrasound study; 18 (32.7%) had metabolic acidosis; 40 (72.7%) received doxycycline, ofloxacin, and ornidazole combination therapy; 15 (27.3%) received doxycycline, ceftriaxone, and metronidazole; and 18 (32.7%) received haemodialysis support.

When cholera patients with AKI and without AKI were compared (Table 1), among 43 cholera patients with AKI, it was noted that the mean age was 34.16 years; the number of males were 30 (69.8%); history of food poisoning was noted in 40 (93%); history of antacid intake was noted in 5 (11.6%); and history of diabetes mellitus was noted in 4 (9.3%) patients. Regarding the severity of diarrhoea, it was noted that severe diarrhoea was noted in 26 (60.5%); moderate diarrhoea was noted in 14 (32.6%); and mild diarrhoea was noted in 3 (7%). Furthermore, it was noted that vomiting was seen in 40 (93%); abdominal pain was noted in 26 (60.5%); fever was noted in 7 (16.3%); blood in stool was noted in 15 (34.9%); the hypovolaemic shock was noted in 40 (93%); severe dehydration was noted in 40 (93%); microscopic haematuria was noted in 19 (44%); mean haemoglobin was 16.7 g%; white blood cell count (WBC) was 10,725/cmm; platelet count was 2.8 lakhs/cmm; mean serum sodium was 129 mEq/L; mean serum potassium was 3.04 meq/L; serum albumin was 3.36 g%; and metabolic acidosis was noted in 18 (41.9%) patients.

Among 12 cholera patients without AKI, it was noted that mean age was 28.58 years; the number of males were 10 (83.3%); history of food poisoning was noted in 11 (91%); history of antacid intake was noted in 1 (8.3%); and history of diabetes mellitus was noted in no patients. Regarding the severity of diarrhoea, it was noted that severe diarrhoea was noted in 5 (41.7%); moderate diarrhoea was noted in 7 (58.3%); and mild diarrhoea was noted in no patients. Furthermore, it was noted that vomiting was seen in 11 (91.7%); abdominal pain was noted in 5 (41.7%); fever was noted in 1 (8.3%); blood in stool was noted in 1 (8.3%); the hypovolaemic shock was noted in 12 (100%); severe dehydration was noted in 12 (100%); microscopic haematuria was noted in no patients; mean haemoglobin was 16.9 g%; white blood cell count (WBC) was 10,375/cmm; platelet count was 2.1 lakhs/cmm; mean serum sodium was 127 mEq/L; mean serum potassium was 3.65 mEq/L; serum albumin was 4 g%; and metabolic acidosis was noted in no patients.

When cholera patients with AKI and without AKI were compared on day 0 and day 5, it was noted that mean serum creatinine on day 0 were 5.7 mg% and 1.16 mg%, respectively (*p* value <0.001) and on day 5 were 1.16 mg% and 1.46 mg%, respectively (*p* value 0.042); mean urine protein-creatinine ratio on day 0 were 1.95 and 0.15, respectively (*p*value 0.002), and on day 5 were 0.31 and 0.18 respectively (*p* value 0.112); and urine output on day 0 were 550 ml/day and 680 ml/day, respectively, and on day 5 were 2,176 ml/day and 2,090 ml/day, respectively.

It was found that there was an insignificant association between hypovolaemic shock (Table 2), severe dehydration (Table 3), presence of diabetes Mellitus between the two groups as shown by Fisher's exact test with the chi-squared value of 0.886, 0.886, and 1.204 and *p* value of 1, 1, and 0.566, respectively.

Mann–Whitney U test was performed to compare the median value of urine PCR (D0) across the groups. It was found to be significant (Mann–Whitney U test statistic value = 112.5 and *p* value = 0.002; Table 4).

Mann–Whitney U test was performed to compare the median value of urine PCR (D5) across the groups. It was found to be not significant (Mann–Whitney U test statistic value = 193 and *p* value = 0.142; Table 4).

There was a good positive correlation between urine PCR (D0) and serum creatinine (D0; Spearman's correlation coefficient = 0.793 and *p* value < 0.001; Table 5).

There was no correlation between urine PCR (D5) and serum creatinine (D5; Spearman's correlation coefficient = 0.298 and *p* value = 0.052; Table 5).

It was noted that doxycycline, ofloxacin, and ornidazole combination therapy was used in 33 (76%) and 7 (58%), respectively, and doxycycline, ceftriaxone, and metronidazole combination therapy was used in 10 (23.3%) and 5 (41%), respectively.

When cholera patients with AKI and without AKI were compared, it was noted that the need for renal replacement therapy was noted in 18 (41.9%) patients and no patients, respectively.

The prognosis was evaluated by looking at the improvement of urine output, serum creatinine, and proteinuria only during the period of admission. Patients were not looked in the postdischarge period as it is a retrospective study, which is mentioned in shortfalls.

## 5. Discussion

Overall, the prevalence of AKI was studied in this part of the world as in our previous study in the same hospital, wherein AKI contributed to 5.2% of all critical care patients, among which 52% needed ICU care. In this study, the prevalence of various AKI stages was not studied in many other previously published studies [6].

AKI was seen in 24.6% of patients. No patients required haemodialysis, and AKI stage 3, AKI stage 2, and AKI stage 1 were seen in 1.8%, 1.8%, and 21%, respectively, though the study group was children [7].

Around 8%–16% of hospital admissions had AKI with 15–16 cases per 1,000 hospitalisations [8–10].

The incidence rate of acute kidney injury (AKI) around the world is not well known. Recent studies have shown that it varies between 4.1 and 288 per 100,000 population in various parts of the world [11].

The incidence of AKI was 51.0%; stages 1–3 accounted for 23.1%, 11.8%, and 15.7%, respectively. A multinational epidemiological study using KDIGO criteria showed that the incidence of AKI in the ICU was 57.3% [12].

Cholera is gastroenteritis caused by *V. cholerae* toxin due to contaminated water and food [1]. The incubation period of cholera is 1–2 days. [1]. The organism was identified using stool hanging drop preparation like other previous studies, for example, Andres Reyes Corcho et al. [1, 13].

Most of our patients had significant vomiting, moderate-to-severe diarrhoea, dehydration, and hypovolaemic shock as specified in the study of Andres Reyes Corcho et al. [1].

Significant hypokalaemia was noted in cholera patients with AKI compared with patients with normal renal function tests as in the study of Sirirat Reungjui et al. [14]. Hyponatraemia did not have any influence. Serum chloride, calcium, and phosphorus were not analysed.

Like in our study, cholera can cause severe complications with severe electrolyte imbalance such as hyponatraemia and hypokalaemia and acute kidney injury (AKI) due to acute tubular necrosis (ATN). Renal complications such as AKI, metabolic acidosis, ATN, acute tubulointerstitial nephritis (ATIN) secondary to dehydration or infection itself have been reported. Renal ischaemia secondary to dehydration is the most common cause of ATN [15]. Metabolic acidosis in cholera is due to loss of bicarbonate in stool and added lactic acidosis due to circulatory collapse and shock [1]. Patients with severe dehydration, prolonged shock, severe haemoconcentration, and hypokalaemia are at higher risk to sustain AKI [1, 15].

A surprising finding noted in our study was the lack of significant association between the onset of AKI and usual risk factors such as hypovolaemic shock and dehydration. Possibly, all these risk factors were appropriately, promptly, and timely treated in our tertiary care hospital.

On further analysis, the following were found:

On day 0, a higher significant median value of urine PCR in the AKI group showed that higher proteinuria was found in that group. There was a strong positive correlation between higher urine PCR and higher serum creatinine in the AKI group, and proteinuria significantly influenced the onset of AKI.

Like a case reported by Basu et al., *Vibrio* infection could have precipitated nephrotic syndrome [16]. Infections are known to cause mesangial proliferative glomerulopathy leading to proteinuria. The temporal presentation of proteinuria, AKI following cholera diarrhoea points towards possible infection associated kidney disease.

On day 5, the insignificant median value of urine PCR across the groups showed that urine PCR resolved to normal value with treatment in the AKI group too. On day 5, there was no correlation between urine PCR and serum creatinine. It showed that improved proteinuria had no significant influence on the resolution of AKI. Our study findings of spontaneous proteinuria remission along with AKI recovery are similar to the study of Han et al. [17] but contrasts with many earlier studies.

Proteinuria is known to damage the kidneys. Nephrotic proteinuria is not normally known to occur in cholera, except as reported in few case reports. In this study, it was noted that higher range proteinuria was found to be in patients with the onset of AKI, possibly hinting that new finding proteinuria could have precipitated AKI in cholera [16, 18, 19].

In this study, usual factors such as severe hypovolaemia and severe dehydration did not influence the onset of AKI, but proteinuria influenced the onset of AKI, unlike in the study of Pierluigi Marzuillo et al. wherein usual factors such as duration of diarrhoea, severe dehydration, and acidosis influenced the onset of AKI in the setting of gastroenteritis in paediatrics though cholera was not included. Therefore, it is difficult to compare both the studies [20].

This indicates that though proteinuria and AKI influenced the onset of each other as specified in the study of Chi-Yuan Hsu et al. [21], it did not influence recovery. It possibly indicates that other factors such as the resolution of dehydration and the hypovolaemic shock would have influenced the recovery of AKI [15]. Hence, as there was complete recovery in kidney function, none of the cases required kidney biopsy. As in our study, spontaneous remission is known to occur in nephrotic syndrome, AKI, and proliferative glomerulonephritis [17]. Like in our study, prompt and timely rehydration and antibiotics reduce AKI incidence in cholera [1, 15, 22]. Like in our study, various antibiotics have been tried to treat cholera in various studies [1, 23, 24].

Like in our study, cholera patients with AKI will require renal replacement therapy [1].

Cholera's morbidity and mortality especially associated with proteinuria are under-reported worldwide [25]. There was no mortality noted in our study group as they were stabilised early by appropriate and prompt early treatment. Generally, mortality is less than 1% in treated cholera patients as specified in the study of Andres Reyes Corcho et al. [1].

### 5.1. Shortfalls

First, this is a retrospective study with a small number of cases being enrolled. The kidney biopsy was not done in any cases to know underlying histopathological changes, which could have added to the pathogenesis and underlying mechanism. The remote possibility of intake of medications such as NSAIDS contributing to this amount of proteinuria can be considered in addition to infection as a cause. Spontaneous remission is known to occur in nephrotic proteinuria, but cases having early remission need to be followed closely. Possible recurrence of proteinuria, the long-term impact of present episodes of proteinuria, AKI, and future new onset renal failure needs to be monitored in all these patients. Though the application of Urine PCR in this kind of setting of AKI where creatinine also varies needs to be ascertained, it has a strong indication for its usage in AKI as specified in the study of Hsu et al. [21]. It is needed to find out whether our study conclusions can be generalised.

## 6. Conclusion

Majority of cholera patients have AKI. Surprisingly, it was noted that there was a lack of significant association between AKI and usual risk factors such as hypovolaemic shock and dehydration. Possibly, all these risk factors were appropriately, promptly, and timely treated in our tertiary care hospital. However, it was found that proteinuria had influenced the onset of AKI, but it did not influence recovery. It possibly indicates that other factors such as the resolution of dehydration and the hypovolaemic shock would have influenced the recovery of AKI. This study points towards the rare occurrence of proteinuria and AKI in *V. cholerae* infection with spontaneous remission of kidney disease with appropriate therapy.

## Figures and Tables

**Table 1:** Comparison between cholera patients with AKI and without AKI.

Variables	Patients with AKI (*n*)	Patients without AKI (*n*)	*p* value
Total patients	43	12	—
Males	30	10	—
Mean age at onset	34	28	—
History of food poisoning	40	11	—
History of long-term antacid intake	5	1	—
Severe diarrhoea	26	5	—
Moderate diarrhoea	14	7	—
Mild diarrhoea	3	0	—
Vomiting	40	11	—
Abdominal pain	26	5	—
Fever	7	1	—
Blood in stool	15	1	—
Hypovolaemic shock	40	12	1
Severe shock	40	12	1
Diabetes mellitus	4	0	0.566
Serum creatinine on day 0 (mg%)	1.16	5.72	<0.001
Serum creatinine on day 5 (mg%)	1.16	1.46	0.042
Serum sodium (mEq/L)	129	127	—
Serum potassium (mEq/L)	3.04	3.65	—
Urine PCR (day 0)	0.15	1.9	0.002
Urine PCR (day 5)	0.18	0.31	0.142
Haemoglobin (g%)	16.9	16.7	—
WBC (/cmm)	10,375	10,725	—
Platelet (L/cmm)	2.1	2.8	—
Microscopic haematuria	19	0	—
Metabolic acidosis	18	0	—
Serum albumin (g%)	4	3.3	—
Urine output D0 (ml/day)	682	552	—
Urine output D5 (ml/day)	2,091	2,176	—
Ceftriaxone + doxycycline + metronidazole	10	5	—
Ofloxacin + doxycycline + ornidazole	33	7	—
Renal replacement therapy	18	0	—

**Table 2:** Correlation of the hypovolaemic shock with cholera.

Cross-tab						
			Group		Total	
			Cholera with normal kidney function	Cholera with abnormal kidney function
Hypovolaemic shock	Yes	Count	12	40	52	
		% within group	100%	93%	94.5%	
	No	Count	0	3	3	
		% within group	0%	7%	5.5%	

Total		Count	12	43	55	
		% within group	100%	100%	100%	

Chi-square Tests						
	Value	*df*	Asymp. sig. (2-sided)	Exact sig. (2-sided)	Exact sig. (1-sided)	Point probability

Pearson chi-squared	0.886^a^	1	0.347	0.587	0.470	0.47
Continuity correction^b^	0.049	1	0.824	—	—	
Likelihood ratio	1.524	1	0.217	0.587	0.470	
Fisher's exact test	—	—	—	1.000	0.470	
Linear-by-linear association	0.869^c^	1	0.351	0.587	0.470	
No. of valid cases	55	—	—	—	—	

**Table 3:** Correlation of dehydration with cholera.

Cross-tab						
			Group		Total	
			Cholera with normal kidney function	Cholera with abnormal kidney function		
Severe dehydration	Yes	Count	12	40	52	
		% within group	100.0%	93.0%	94.5%	
	No	Count	0	3	3	
		% within group	0.0%	7.0%	5.5%	

Total		Count	12	43	55	
		% within group	100.0%	100.0%	100.0%	

Chi-squared tests						
	Value	df	Asymp. sig. (2-sided)	Exact sig. (2-sided)	Exact sig. (1-sided)	Point probability

Pearson's chi-squared	0.886^a^	1	0.347	0.587	0.470	0.470
Continuity correction^b^	0.049	1	0.824	—	—	
Likelihood ratio	1.524	1	0.217	0.587	0.470	
Fisher's exact test	—	—	—	1.000	0.470	
Linear-by-linear association	0.869^c^	1	0.351	0.587	0.470	
No. of valid cases	55	—	—	—	—	

**Table 4:** Correlation of proteinuria (urine PCR) with cholera.

Ranks				
	Group	*N*	Mean rank	Sum of ranks

Urine PCR (D0)	Cholera with normal kidney function	12	15.88	190.50
	Cholera with abnormal kidney function	43	31.38	1,349.50
	Total	55	—	—

Urine PCR (D5)	Cholera with normal kidney function	12	22.58	271.00
	Cholera with abnormal kidney function	43	29.51	1,269.00
	Total	55	—	—

Test statistics^a^				
	Urine PCR (D0)		Urine PCR (D5)	

Mann–Whitney U	112.500		193.000	
Wilcoxon W	190.500		271.000	
*Z*	−3.053		−1.468	
Asymp. sig. (2-tailed)	0.002		0.142	

**Table 5:** Correlation between proteinuria (urine PCR) with renal failure (serum creatinine).

Correlations				
			Urine PCR (D0)	Serum creatinine (D0; mg/dl)

Spearman's rho	Urine PCR (D0)	Correlation coefficient	1.000	0.793^*∗∗*^
		Sig. (2-tailed)	—	0.000
		*N*	43	43
	Serum creatinine (D0; mg/dl)	Correlation coefficient	0.793^*∗∗*^	1.000
		Sig. (2-tailed)	0.000	—
		*N*	43	43

Correlations				
			Urine PCR (D5)	Serum creatinine (D5; mg/dl)

Spearman's rho	Urine PCR (D5)	Correlation coefficient	1.000	0.298
		Sig. (2-tailed)	—	0.052
		*N*	43	43
	S. creatinine (mg/dl) D5	Correlation coefficient	0.298	1.000
		Sig. (2-tailed)	0.052	—
		*N*	43	43

## Data Availability

The data that support the findings in this study are included in the article itself.
